# Different levels of cardiometabolic indicators in multiple vs. singleton children

**DOI:** 10.1186/s12887-019-1707-0

**Published:** 2019-09-11

**Authors:** Maria João Fonseca, Ana Cristina Santos, Henrique Barros

**Affiliations:** 1 ISPUP-EPIUnit, Universidade do Porto, Rua das Taipas n° 135, 4050-600 Porto, Portugal; 2 Departamento de Ciências da Saúde Pública e Forenses e Educação Médica, Faculdade de Medicina, Universidade do Porto, Al. Prof. Hernâni Monteiro, 4200-319 Porto, Portugal

**Keywords:** Anthropometric measures, Birthweight, Body composition, Cardiometabolic, Twins

## Abstract

**Background:**

We aimed to compare cardiometabolic indicators in singletons and multiples at age 7 and explore the birthweight mediation effect.

**Methods:**

We studied 5431 singletons and 103 sets of multiples from Generation XXI birth cohort. Anthropometric measurements, body composition, and fasting blood samples were obtained. Age- and sex-specific z-scores were calculated (additionally height-specific for blood pressure). Adjusted regression coefficients and respective 95% confidence intervals [β (95%CI)] were computed using path analysis.

**Results:**

Multiples had lower weight [− 0.419 (− 0.616;-0.223)], height [− 0.404 (− 0.594;-0.213)], BMI [− 0.470 (− 0.705;-0.234)], fat mass index [− 0.359 (− 0.565;-0.152)], waist circumference [− 0.342 (− 0.537;-0.147)], and waist-to-height ratio [− 0.165 (− 0.326;-0.003)] z-scores. These results were explained by the indirect effect via birthweight, which was also negative and significant for all the aforementioned cardiometabolic indicators, while no direct effect was present. There were also significant indirect effects regarding fat-free mass index, glucose, insulin, and blood pressure, though the total effects were not significant, due to the balance between direct and indirect effects. The only significant direct effect was regarding diastolic blood pressure [− 0.165 (− 0.302;-0.028)].

**Conclusions:**

At age 7, multiples presented better cardiometabolic indicators explained by lower weight at birth, except for the lower blood pressure which was independent of an effect via birthweight.

**Electronic supplementary material:**

The online version of this article (10.1186/s12887-019-1707-0) contains supplementary material, which is available to authorized users.

## Background

An offspring of a multiple pregnancy differs from a singleton regarding characteristics such as fetal growth [1, 2], gestational age [1, 3], mode of delivery [3, 4], anthropometric measures at birth [1, 3, 4] and complications during the neonatal period [1, 4].

Usually, multiples present a lower birthweight, due to intrauterine growth restriction [1, 2] and lower gestational age [1, 3]. Low birthweight is associated with increased risk of neonatal morbidity and mortality, but also with adverse long-term consequences, namely those related with early cardiometabolic programming [5]. Yet, studies performed in singletons showed that either low or high birthweight are associated with obesity and other unfavourable cardiometabolic markers [5–8]. Moreover, the particular intrauterine growth circumstances of multiples might impact on later health [2]. Considering neonatal prognostic, optimal birthweight seems to be lower for multiple babies than for singletons [9], yet this effect seems to disappear when adjusted for relative birthweight [10]. The same may not hold true for long-term outcomes.

In addition to a slower rate of growth both in utero [1, 2] and during the first years of life [11, 12], multiple babies experience particular circumstances [13] (e.g.: sharing the utero and nutrition during intra uterine life, sharing the mother’s and father’s attention, having a same-age brother/sister), which might be influential throughout life.

Thus, we can hypothesize that multiples conserve particular anthropometric parameters during childhood and different levels of cardiometabolic indicators. Yet, to the best of our knowledge, no study has so far evaluated the effect of being multiple on cardiometabolic indicators during childhood. Thus, we compared anthropometric, body composition, serum cardiometabolic markers, and blood pressure in multiples and singletons at 7 years of age and explored the mediation effect of birthweight.

## Methods

Participants of the present study are part of the Generation XXI birth cohort [14]. Between April 2005 and August 2006, mothers given birth at one of the five level III public units providing obstetrical and neonatal care in the metropolitan area of Porto, Portugal, were consecutively invited to participate. All the maternities, except one, were included in a general hospital, with a variety of medical and surgical specialties, and all corresponded to level III maternity units, with differentiated perinatal support. At birth, 91.4% of the invited mothers accepted to participate. A total of 8495 mothers, who gave birth to 8647 live born infants, which included 137 twin pairs, 6 triplets, 1 quadruplet, and 8351 singletons, were enrolled into the cohort. At 7 years of age, all cohort members were invited to be re-evaluated (April 2012 to March 2014), and 6889 (80%) agreed to that follow-up.

At recruitment, information on maternal socio-demographic, behavioural and pre-pregnancy anthropometric characteristics was collected by face-to-face interview using structured questionnaires, completed 24 to 72 h after delivery, during the hospital stay. Data on delivery and newborn characteristics were additionally abstracted from clinical records, as previously described [7, 14].

At 7 years of age, child’s anthropometric measurements were performed by trained examiners, according to standard procedures, as previously described [7]. Waist-to-height ratio (WHtR) was calculated as WHtR = Waist Circumference (WC)(cm)/height(cm). Child’s Body Mass Index (BMI) was calculated as BMI = weight (kg)/height ^2^ (m) and age- and sex-specific z-scores were established according to the World Health Organization [15]. Body composition was measured by a bioelectrical impedance analysis generator [7] and following the approach proposed by Horlick et al. [16], we previously tested all equations considered by the author, and chose the one proposed by Schaefer et al. [17]. Fat mass (FMI) and fat-free mass indexes (FFMI) were calculated as FMI = total fat (kg)/height^2^ (m) and FFMI = total fat-free mass (kg)/height^2^ (m). Age- and sex-specific z-scores were established for weight, height, WC, WHtR, FMI and FFMI based on the age- and sex-specific means and standard deviations (SD) derived from the whole cohort.

Two measurements of blood pressure, separated by at least 5 min, were taken after 10-min rest. If the difference between the two individual measurements was lower than 5 mmHg, the mean was recorded, if larger than 5 mmHg a third measurement was taken and the mean of the 2 closest values was used. For systolic (SBP) and diastolic (DPB) blood pressure, age-, sex- and height-specific z-scores were calculated, following the recommendations of the American Academy of Pediatrics [18].

After an overnight fast, a venous blood sample was drawn, after applying a topical analgesic cream (EMLA cream), centrifuged at 3500 rpm for 10 min, aliquoted, immediately analysed or deep frozen until analysis. Glucose was measured using an UV enzymatic assay (hexokinase method), insulin using electrochemiluminescence immunoassay, HDL-cholesterol and triglycerides (TG) using enzymatic colorimetric assays, and high-sensitivity C-reactive protein (hs-CRP) using nephelometry. Age- and sex-specific z-scores were computed based on the age- and sex-specific means and standard deviations (SD) derived from the whole Generation XXI cohort.

The cardiometabolic traits analyzed included anthropometrics, body composition, serum cardiometabolic markers, and blood pressure to be as much comprehensive as possible. Concerning serum cardiometabolic markers we chose to include those present in the definition of the metabolic syndrome (glucose, TG, and HDL-cholesterol) and added insulin and hs-CRP as extra markers of glucose metabolism and inflammatory process, respectively.

At 7 years of age, 212 multiples and 5431 singletons had a physical exam and provided blood samples. The comparison between these participants and the remaining eligible children, stratified by singleton/multiple status, is shown in Table 1. Mothers of singleton participants were older, more educated, more frequently were married or living with a partner, were primiparae, had been more frequently submitted to fertility treatment, had a higher prevalence of caesarean delivery, and less frequently smoked during the third pregnancy trimester. Among multiples, participant mothers were more educated, the babies presented higher birthweight, and a higher frequency of monozygotic twins.

**Table 1:** Maternal, pregnancy, delivery and newborn characteristics of participants and eligible non participants, stratified by singleton/multiple status

	Singletons			Multiples		
	Participants	Non participants	p	Participants	Non participants	p
	*n* = 5431	*n* = 2920		*n* = 212 (103 sets)	*n* = 84 (41 sets)	
Maternal characteristics						
Maternal education (years), mean (SD)	11.0 (4.3)	9.4 (4.0)	< 0.001	11.4 (4.5)	9.7 (4.4)	0.043
Maternal age (years), mean (SD)	29.7 (5.3) ^a^	27.7 (5.9)	< 0.001	30.5 (4.1) ^a^	29.6 (5.4)	0.316
Pre-pregnancy BMI (kg/m^2^), mean (SD)	23.9 (4.2)	23.8 (4.5)	0.435	23.7 (4.0)	24.8 (3.7)	0.132
Marital status, n (%)						
Married/Living with a partner	5126 (94.9)	2638 (91.1)		99 (97.1)	41 (100.0)	
Single/Widowed/Divorced/Separate	273 (5.1)	259 (8.9)	< 0.001	3 (2.9)	0 (0.0)	0.557 ^b^
Parity, n (%)						
Multiparae	2219 (40.9)	1334 (45.7)		34 (33.3)	13 (31.7)	
Primiparae	3212 (59.1)	1586 (54.3)	< 0.001	68 (66.7)	28 (68.3)	0.852
Pregnancy and delivery characteristics						
Fertility treatment, n (%)	110 (2.1) ^a^	31 (1.1)	0.001	23 (23.0) ^a^	5 (12.2)	0.144
Gestational diabetes, n (%)	339 (6.3)	175 (6.1)	0.662	5 (4.9)	3 (7.5)	0.687 ^b^
Hypertension, n (%)	131 (2.4)	64 (2.2)	0.531	4 (3.9)	0 (0.0)	0.577 ^b^
Pre-eclampsia / Eclampsia, n (%)	57 (1.1) ^a^	42 (1.5)	0.116	5 (4.9) ^a^	3 (7.5)	0.546 ^b^
Tobacco smoke during 3rd trimester, n (%)	629 (11.9)	542 (19.2)	< 0.001	6 (5.9)	5 (13.2)	0.172 ^b^
Weight gain (kg)	12.8 (5.3) ^a^	12.7 (5.7)	0.520	14.8 (6.5) ^a^	12.8 (6.8)	0.136
Gestational age (weeks), mean (SD)	38.7 (1.7) ^a^	38.6 (1.9)	0.077	34.7 (3.0) ^a^	34.1 (3.1)	0.319
Mode of delivery, n (%)						
Vaginal	3362 (63.1) ^a^	1933 (67.5)		29 (29.9) ^a^	12 (29.3)	
Caesarean	1967 (36.9) ^a^	931 (32.5)	< 0.001	68 (70.1) ^a^	29 (70.7)	0.941
Newborn characteristics						
Triplets, n (%)	–	–	n.a.	12 (5.8)	6 (7.1)	0.659
Monozygotia, n (%)	–	–	n.a.	31 (31.3)	3 (11.1)	0.036
Sex, n (%)						
Male	2798 (51.5)	1468 (50.3)		105 (50.5) ^c^	38 (45.2) ^c^	
Female	2633 (48.5)	1452 (49.7)	0.278	103 (49.5) ^c^	46 (57.8) ^c^	0.493
Birthweight (g), mean (SD)	3193 (488) ^a^	3173 (509)	0.090	2152 ^ac^	2032 ^c^	< 0.001
Birthweight for gestational age						
Z-score, mean (SD)	−0.31 (0.87) ^a^	-0.31 (1.1)	0.897	− 0.83 ^ac^	− 0.83 ^c^	0.996
Small for gestational age, n (%)	675 (12.5) ^a^	387 (13.4)		60 (29.3) ^ac^	23 (27.4) ^c^	
Normal for gestational age, n (%)	4542 (84.0) ^a^	2372 (82.2)		143 (69.8) ^ac^	60 (71.4) ^c^	
Large for gestational age, n (%)	189 (3.5) ^a^	126 (4.4)	0.056	2 (1.0) ^ac^	1 (1.2) ^c^	0.845 ^b^
Phototherapy treatment, n (%)	659 (13.3) ^a^	349 (13.3)	0.979	64 (39.5) ^ac^	41 (57.7) ^c^	0.055
NICU admission, n (%)	314 (6.4) ^a^	209 (8.0)	0.011	91 (52.6) ^ac^	40 (57.1) ^c^	0.722
APGAR 5′ < 7, n (%)	29 (0.6) ^a^	15 (0.5)	0.906	7 (3.8) ^a c^	4 (4.9) ^c^	0.700

^a^ Significant differences (p < 0.05) between singleton participants and multiple participants

^b^ Fisher exact test

^c^ Adjusted for non independence

All the phases of the study complied with the Ethical Principles for Medical Research Involving Human Subjects expressed in the Declaration of Helsinki. The study was approved by the University of Porto Medical School/ S. João Hospital Centre Ethics Committee and parents or legal representatives signed an informed consent. The study was approved by the Portuguese Data Protection Authority.

### Statistical analysis

Proportions were compared using the chi-square test or Fisher exact test whenever adequate, and means were compared using student t-test (analysis performed using SPSS version 23.0).

Linear regression coefficients (β) and 95% confidence intervals (95% CI) were computed using path analysis. Since this method does not allow adjustment for non-independence, a random child was chosen from each set of twins to perform the path analysis. The fit of the models was assessed using different indexes: the Comparative Fit Index (CFI), the Tucker–Lewis Index (TLI), and the Root Mean Square Error of Approximation (RMSEA). A good model fit is indicated by a CFI and TLI values ≥0.90 and values of RMSEA lower than 0.05.

We defined confounders as events occurring prior to conception that were associated with both singleton/multiple status and the outcomes and mediators as post-conception events that were associated both with singleton/multiple status and the outcomes, being an intermediate step in the hypothesized causal chain [7]. Maternal age, education, pre-pregnancy BMI, marital status, parity, and fertility treatment were tested as potential confounders. Diabetes and hypertension during pregnancy, pre-eclampsia/eclampsia, gestational age, mode of delivery, birthweight, and birthweight for gestational age [19] were tested as potential mediators. Of those variables, only maternal age, fertility treatment, gestational age, birthweight, and birthweight for gestational age were significantly associated with both singleton/multiple status and at least one of the outcomes. We found strong correlations between gestational age, birthweight, and birthweight for gestational age. However, of these three potential mediators, birthweight presented the strongest association with the outcomes. Also, interactions with birthweight, sex and mode of delivery were tested and not found.

Accordingly, the confounders included in the models were maternal age (years) and fertility treatment (yes vs. no) and the mediator included was birthweight (decigram). Path analysis was performed for each of the outcomes (z-scores) with Mplus on R software [20]; 95% confidence intervals were calculated by bootstrapping.

## Results

Mean maternal age (30.5 vs. 29.7 years, *p* = 0.043) and the frequency of fertility treatment (23.0% vs. 2.1%, *p* < 0.001) were higher among multiples (Table 1). Considering the potential mediators, multiples had higher proportion of pre-eclampsia/eclampsia during pregnancy (4.9% vs. 1.1%, *p* = 0.006), lower mean gestational age (34.7 vs. 38.7 weeks, *p* < 0.001), higher proportion of caesarean delivery (70.1% vs. 36.9%, *p* < 0.001), lower mean birthweight (2152 g vs. 3193 g, *p* < 0.001) and lower z-scores of birthweight for gestational age (− 0.83 vs. − 0.31, *p* < 0.001) than singletons (Table 1).

As observed in Table 2, multiples had lower mean weight (24.0 vs. 26.2 kg, *p* < 0.001), height (121.3 vs. 123.6 cm, *p* < 0.001), BMI (16.3 vs. 17.1 kg/m^2^, *p* < 0.001), FMI (2.3 vs. 3.0 kg/m^2^, *p* = 0.001), WC (56.6 vs. 59.1 cm, *p* < 0.001), WHtR (0.47 vs. 0.48, *p* = 0.023), SBP (103.4 vs. 105.2 mmHg, *p* = 0.016) and DBP (68.1 vs. 69.8 mmHg, *p* = 0.006) than singletons. When comparing the z-scores of the aforementioned cardiometabolic indicators, the results were similar, except for SBP and DBP z-scores, which were similar in singletons and multiples. As a sensitivity analysis, the same comparison was performed restricting the analysis only to small for gestational age children (Additional file 1: Table S1) and the results were similar. Small for gestational age multiples had significantly lower mean BMI, FFMI, WC, WHtR, SBP and DBP than small for gestational age singletons. Additionally, also as a sensitivity analysis, a matched pair analysis, matching each multiple with the singleton with the closest gestational age, was performed and all the results were in the same direction - multiples presented lower z-scores of weight, height, BMI, FMI, WC, and WHtR - significant only for weight, height and WC (Additional file 2: Table S2).

**Table 2:** Comparison between singletons and multiples regarding cardiometabolic indicators at 7 years follow-up evaluation

	Crude value		p	Age and sex z-score^b^		p
	Singletons	Multiples		Singletons	Multiples	
Cardiometabolic characteristics at age 7^a^						
Weight (kg)	26.2 (26.1, 26.4)	24.0 (23.1, 25.0)	< 0.001	0.02 (−0.01; 0.04)	− 0.41 (− 0.59; − 0.24)	< 0.001
Height (cm)	123.6 (123.4, 123.7)	121.3 (120.3, 122.2)	< 0.001	− 0.01 (− 0.04; 0.02)	− 0.45 (− 0.62; − 0.28)	< 0.001
Body mass index (kg/m^2^)	17.1 (17.0, 17.1)	16.3 (15.8, 16.7)	< 0.001	0.73 (0.70; 0.76)	0.27 (0.07; 0.48)	< 0.001
Fat mass index (kg/m^2^)	3.0 (2.9, 3.1)	2.3 (1.9, 2.7)	0.001	−0.07 (− 0.10; − 0.04)	−0.39 (− 0.57; − 0.21)	0.001
Fat-free mass index (kg/m^2^)	14.0 (14.0, 14.1)	13.9 (13.7, 14.2)	0.360	0.09 (0.07; 0.12)	0.01 (− 0.16; 0.18)	0.325
Waist circumference (cm)	59.1 (59.0, 59.3)	56.6 (55.4, 57.8)	< 0.001	−0.03 (− 0.06; − 0.01)	−0.40 (− 0.57; − 0.23)	< 0.001
Waist-to-height ratio	0.48 (0.48, 0.48)	0.47 (0.46, 0.48)	0.023	−0.05 (− 0.07; − 0.03)	−0.20 (− 0.34; − 0.06)	0.044
Glucose (mg/dL)	82.4 (82.2, 82.7)	82.4 (80.7, 84.1)	0.980	−0.09 (− 0.13; − 0.05)	− 0.11 (− 0.40; 0.19)	0.909
Insulin (μIU/mL)	5.2 (5.0, 5.3)	4.5 (3.8, 5.2)	0.061	0.01 (− 0.03; 0.04)	− 0.20 (− 0.43; 0.03)	0.082
HDL-cholesterol (mg/dL)	56.3 (55.9, 56.7)	58.0 (55.4, 60.6)	0.201	0.08 (0.04; 0.11)	0.22 (−0.01; 0.44)	0.218
Triglycerides (mg/dL)	62.2 (61.3, 63.2)	60.9 (54.8, 67.1)	0.693	−0.05 (− 0.09; − 0.02)	−0.09 (− 0.29; 0.12)	0.745
hs-C-reactive protein (mg/L)	1.31 (1.20, 1.43)	1.02 (0.43, 1.61)	0.346	0.00 (− 0.03; 0.04)	− 0.03 (− 0.28; 0.22)	0.816
Systolic blood pressure (mmHg)	105.2 (105.0, 105.5)	103.4 (102.0, 104.9)	0.016	0.71 (0.68; 0.73)	0.70 (0.58; 0.83)	0.970
Diastolic blood pressure (mmHg)	69.8 (69.7, 70.1)	68.1 (67.0, 69.4)	0.006	1.04 (1.02; 1.06)	0.97 (0.86; 1.07)	0.185

^a^ Mean values and 95% confidence intervals adjusted for non independence

^b^ For systolic and diastolic blood pressure - age, sex and height z-score

Table 3 shows the total, direct and indirect effect of multiple status on cardiometabolic indicators z-scores at 7 years of age, resulting from the path analysis. Multiples had lower age and sex z-scores for weight [− 0.419 (− 0.616; − 0.223)], height [− 0.404 (− 0.594; − 0.213)], BMI [− 0.470 (− 0.705; − 0.234)], FMI [− 0.359 (− 0.565; − 0.152)], WC [− 0.342 (− 0.537; − 0.147)], and WHtR [− 0.165 (− 0.326; − 0.003)]. These total effects were explained by the indirect effect via birthweight, which was also negative and significant for all the previous mentioned cardiometabolic indicators, while no direct effect was present. There were also significant indirect effects regarding FFMI, glucose, insulin, SBP and DBP, though the total effect was not significant, due to the balance between the direct and indirect effects. The only significant direct effect observed was for DBP [− 0.165 (− 0.302; − 0.028)].

**Table 3:** Total, direct and indirect effect of multiple status (vs. singleton status) on cardiometabolic indicators z-scores at 7 years of age

	β [95% CI] in Multiples ^a^		
	Total effect	Direct effect	Indirect effect
Age and sex z-scores ^b^			
Weight	−0.419 (− 0.616; − 0.223)*	−0.020 (− 0.221; 0.181)	−0.399 (− 0.466; − 0.333)*
Height	−0.404 (− 0.594; − 0.213)*	0.083 (− 0.110; 0.275)	−0.486 (− 0.556; − 0.416)*
Body mass index	−0.470 (− 0.705; − 0.234)*	−0.133 (− 0.376; 0.109)	−0.336 (− 0.409; − 0.263)*
Fat mass index	−0.359 (− 0.565; − 0.152)*	−0.109 (− 0.322; 0.104)	−0.249 (− 0.312; − 0.187)*
Fat-free mass index	−0.057 (− 0.259; 0.145)	0.019 (− 0.192; 0.229)	−0.075 (− 0.133; − 0.018)*
Waist circumference	−0.342 (− 0.537; − 0.147)*	−0.086 (− 0.287; 0.115)	−0.256 (− 0.315; − 0.196)*
Waist-to-height ratio	−0.165 (− 0.326; − 0.003)*	−0.096 (− 0.264; 0.072)	−0.069 (− 0.115; − 0.023)*
Glucose	0.218 (− 0.117; 0.553)	0.300 (− 0.044; 0.644)	−0.082 (− 0.160; − 0.004)*
Insulin	−0.141 (− 0.419; 0.137)	−0.072 (− 0.358; 0.213)	−0.069 (− 0.133; − 0.004)*
HDL-cholesterol	0.148 (− 0.112; 0.408)	0.122 (− 0.145; 0.389)	0.026 (− 0.034; 0.086)
Triglycerides	−0.014 (− 0.254; 0.226)	−0.004 (− 0.250; 0.243)	−0.010 (− 0.066; 0.045)
hs-C-reactive protein	− 0.093 (− 0.374; 0.188)	−0.107 (− 0.396; 0.182)	0.014 (− 0.051; 0.079)
Systolic blood pressure	0.057 (− 0.103; 0.217)	−0.056 (− 0.222; 0.110)	0.113 (0.066; 0.159)*
Diastolic blood pressure	−0.064 (− 0.196; 0.068)	−0.165 (− 0.302; − 0.028)*	0.100 (0.062; 0.139)*

*p < 0.05

^a^ Reference category: singletons

^b^ For systolic and diastolic blood pressure - age, sex and height z-score

**Fig. 1:**
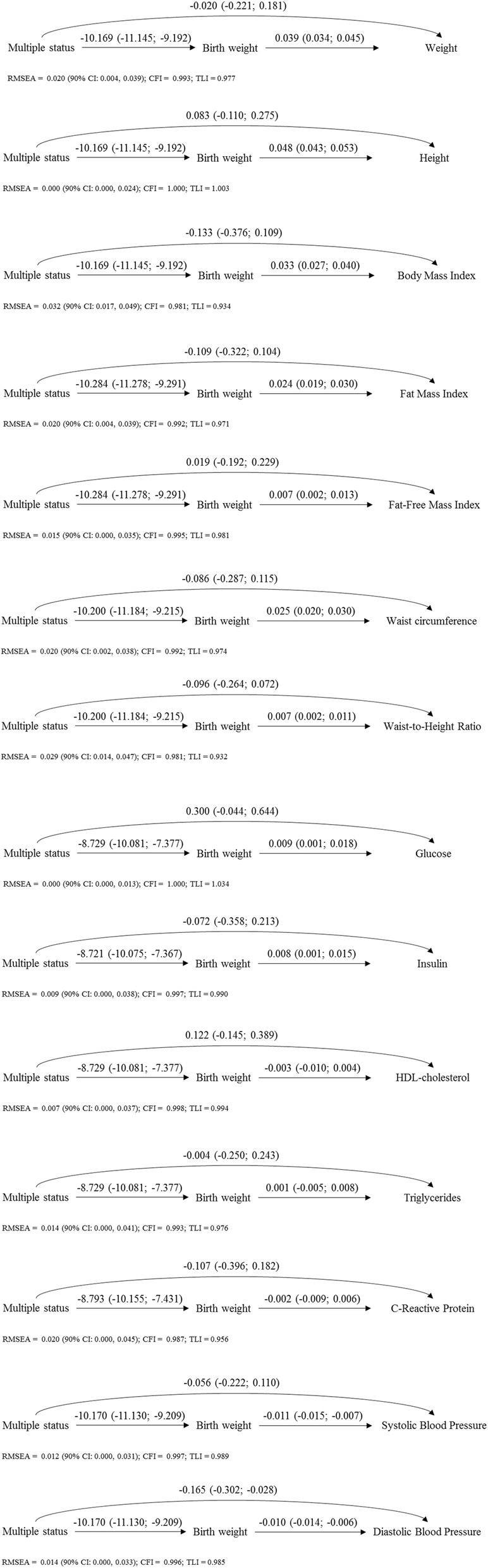
Path analysis of the associations of multiple status (vs. singleton status) on cardiometabolic indicators z-scores at 7 years of age

Figure 1 presents the regression coefficients and 95% confidence intervals [β (95% CI)] for the different paths of the associations. In this figure, the indirect effects can be observed, comprising an inverse association between multiple status and birthweight plus: a) a positive association between birthweight and weight, height, BMI, FMI, FFMI, WC, WHtR, glucose and insulin; b) an inverse association between birthweight and SBP and DBP.

## Discussion

In this study, we compared multiples and singletons regarding cardiometabolic indicators at age 7. We found that multiples presented lower adiposity, with significantly lower adjusted weight, height, BMI, FMI, and central adiposity. These differences were explained by their lower birthweight. Multiples had also lower blood pressure, after removing the effect via birthweight. Multiples and singletons were similar regarding FFMI, glucose, insulin, HDL-cholesterol, triglycerides, and hs-CRP levels.

Multiples were shorter, lighter, and thinner than singletons. The lower BMI was largely explained by a lower FMI - 0.359 standard deviations lower in multiples. In addition, multiples had lower WC and WHtR, meaning less central adiposity. After removing the effect via birthweight, the observed associations were attenuated towards zero, no direct effect being present. In contrast, the indirect effect (via birthweight) was significant for all those variables, which means that multiples presented lower estimates for anthropometric and body composition measures reflecting mostly their lower birthweight. Multiples have lower birthweight [1–3], which in turn has been associated with lower anthropometric and body composition measurements later in life, in several recent cohorts [6, 21, 22], including Generation XXI [7]. This is explained by the tracking effect, and in this regard, literature shows that BMI and fatness tracks from early life until adulthood [23, 24]. On the other hand, it is known that catch-up growth, the acceleration in growth that occurs when a period of growth retardation ends and favourable conditions are restored [25], usually occurs in children that are born with lower birthweight, which is the case of multiples. However, data from the third National Health and Nutrition Examination Survey (NHANES) and the National Collaborative Perinatal Project showed that despite the catch-up growth, children that were born with lower birthweight remained shorter and lighter through childhood [26, 27], which may explain why multiples remain smaller than singletons during childhood. Sensitivity analysis were performed taking into consideration other potential mediators and none fully explained the lower anthropometric and body composition measures, except for birthweight.

Concerning blood pressure, the levels of sex-, age- and height- specific z-scores were similar in multiples and singletons. Similarly, a study using data from the Netherlands Twin Registry found that multiples and their singleton siblings had similar blood pressure levels in adulthood [28]. Nevertheless, when the mediator effect via birthweight was removed, a direct association between multiple status and lower blood pressure emerged, significant for DBP. Lower birthweight has been described as a risk factor for higher blood pressure [29], this being partly explained by decreased number of nephrons in lower birthweight children [29, 30], suggesting that multiples could have higher blood pressure than singletons, merely due to their lower birthweight (i.e. a positive birthweight-mediated association, which actually occurred in the present study, since the indirect effect was positive and significant for SBP and DBP). However, when analyzing the association of singleton/multiple status with blood pressure independently of this birthweight-mediated effect, multiple status was directly associated with lower blood pressure (i.e. for a given fixed birthweight, multiples would have lower blood pressure than singletons). We further tested adjustment for other possible mediators – current BMI and current weight – but none explained the association. So, other mechanisms than the attained weight must be involved in this direct association of multiple status and lower blood pressure. One of those mechanisms could be the hypothesis of in utero programming, where a different in utero development of kidneys or of hypothalamic-pituitary-adrenal-axis makes possible that for a given birthweight multiples present a lower blood pressure than singletons. It had already been shown in animal models that despite multiples do have a lower number of nephrons, they did not present higher blood pressure levels [30]. Our data needs to be replicated in different samples to clarify why multiples, despite having lower birthweight, do not present higher blood pressure later in life and may even have lower blood pressure when analyzing independently of birthweight.

At the age of 7, blood levels of glucose, insulin, HDL-cholesterol, triglycerides, and hs-CRP were similar in multiples and singletons. Since multiplicity is relatively uncommon, it would be possible that these findings reflect lack of statistical power. However, given that most point estimates were close to the null, this does not seem probable.

### Limitations and strengths

This study includes a population-based sample and is the first to examine the effect of singleton/multiple status on cardiometabolic indicators in childhood. Although statistically significant differences between participants and non-participants existed, the magnitude of those differences were small, not expecting to have influenced the conclusions.

At 7 years of age, information was obtained using measurements performed by trained health professionals according to standard procedures, which preclude recall bias. Although information on a 24-h blood pressure assessment were not available, we believe that the blood pressure measured on a single occasion was capable of discriminate children.

The low number of multiples, which is expected in a general population-based study, increases the chance of type II error. Nevertheless, associations between multiple status and anthropometric and body composition parameters and also blood pressure were detected. However, it is possible that type II error occurred when analyzing blood parameters.

Sensitivity analyses were performed – one restricting the analysis only to small for gestational age children and the other matching each multiple with the singleton with the closest gestational age (matched pair analysis) – in order to explore, to some extent, the role of these two potential mediators. The results were in the same direction, although some lost statistical significance or were attenuated, which was expected because when we take into account gestational age or birthweight for gestational age, we are inevitably taking birthweight into account. Additionally, in the matched pair analysis, DBP appeared to be lower in multiples, which also occurred when we adjusted for birthweight. So, we believe these results improve the level of evidence of our main findings.

The present study is one of the few to evaluate cardiometabolic indicators this early in life. As abnormal values of cardiometabolic indicators are unusual at these ages, it is possible that the medium- and/or long-term effects of multiple/singleton status on cardiometabolic indicators are not yet fully evident and, with aging, stronger long-term effects might appear. Still, a multiple/singleton status effect on anthropometric and body composition measures and blood pressure seemed evident.

Even though birthweight explained most of the association between multiple status and cardiometabolic traits, we are aware that other variables such as gestational age, immaturity or mode of delivery could also play a role. Yet, given the multicollinearity, when taking into account the mediator birthweight, we are also taking into account part of such variables.

Despite the limitations of the present study, we believe that our results demonstrate how multiples and singletons differ in terms of cardiometabolic indicators at 7 years of age and generate the hypothesis of an explanation via birthweight. Yet, these results should be replicated in other populations and other mechanisms explaining the differences found between multiples and singletons must be explored.

## Conclusions

At age 7, multiples presented lower total and central adiposity explained by a lower weight at birth. Multiple status also affect glucose, insulin and SBP, but only indirectly via birthweight, i.e. multiples had lower birthweight which in turn led to lower glucose and insulin levels and higher SBP.

## Additional files

Additional file 1: **Table S1.** Comparison between small for gestational age singletons and small for gestational age multiples regarding cardiometabolic indicators at 7 years follow-up evaluation. (DOCX 16 kb)

Additional file 2: **Table S2.** Matched pair analysis regarding the comparison between singletons and multiples regarding cardiometabolic indicators at 7 years follow-up evaluation. (DOCX 14 kb)

## Data Availability

The datasets used and/or analysed during the current study are available from the corresponding author on reasonable request.

## Abbreviations

BMI: body mass index
DBP: diastolic blood pressure
FFMI: fat-free mass index
FMI: Fat mass index
hs-CRP: high-sensitivity C-reactive protein
LDL-C: low density lipoprotein cholesterol
SBP: systolic blood pressure
TG: triglycerides
WC: Waist Circumference
WHtR: Waist-to-height ratio
