# Supplementary material for: Different levels of cardiometabolic indicators in multiple vs. singleton children
Source: BMC Pediatr. 2019 Sep 11;19:331. doi: 10.1186/s12887-019-1707-0 (PMC6737661; doi:10.1186/s12887-019-1707-0)
Supplement: Supplementary file 1 — Table S1. Comparison between small for gestational age singletons and small for gestational age multiples regarding cardiometabolic indicators at 7 years follow-up evaluation. (DOCX 16 kb) [file 12887_2019_1707_MOESM1_ESM.docx]

| Supplementary table 1. Comparison between small for gestational age singletons and small for gestational age multiples regarding cardiometabolic indicators at 7 years follow-up evaluation. | | | | | | |
| --- | --- | --- | --- | --- | --- | --- |
|  |  |  |  |  |  |  |
|  | **Crude value** | |  | **Age and sex z-score^b^** | |  |
|  | **Singletons** | **Multiples** | **p** | **Singletons** | **Multiples** | **p** |
| **Cardiometabolic characteristics at age 7^a^** |  |  |  |  |  |  |
| Weight (kg) | 24.6 (24.2, 25.0) | 23.4 (21.9, 24.8) | 0.104 | -0.30 (-0.37; -0.23) | -0.56 (-0.83; -0.28) | 0.074 |
| Height (cm) | 121.3 (120.9, 121.7) | 122.0 (120.5, 123.6) | 0.393 | -0.43 (-0.51; -0.36) | -0.37 (-0.65; -0.09) | 0.675 |
| Body mass index (kg/m^2^) | 16.6 (16.4, 16.8) | 15.6 (14.9, 16.3) | 0.007 | 0.48 (0.38; 0.57) | -0.09 (-0.45; 0.26) | 0.002 |
| Fat mass index (kg/m^2^) | 2.6 (2.4, 2.8) | 2.0 (1.3, 2.7) | 0.080 | -0.23 (-0.31; -0.16) | -0.53 (-0.82; -0.24) | 0.053 |
| Fat-free mass index (kg/m^2^) | 14.0 (13.9, 14.1) | 13.6 (13.2, 14.0) | 0.038 | 0.04 (-0.04; 0.12) | -0.33 (-0.64; -0.03) | 0.021 |
| Waist circumference (cm) | 57.7 (57.2, 58.2) | 55.6 (53.6, 57.6) | 0.043 | -0.23 (-0.30; -0.16) | -0.58 (-0.86; -0.30) | 0.020 |
| Waist-to-height ratio | 0.48 (0.47, 0.48) | 0.46 (0.44, 0.47) | 0.006 | -0.08 (-0.14; -0.01) | -0.40 (-0.63; -0.16) | 0.011 |
| Glucose (mg/dL) | 83.3 (82.7, 83.9) | 83.5 (81.2, 85.8) | 0.873 | 0.06 (-0.05; 0.17) | 0.08 (-0.31; 0.47) | 0.924 |
| Insulin (μIU/mL) | 5.9 (5.4, 6.4) | 4.1 (2.2, 5.9) | 0.057 | 0.27 (0.11; 0.42) | -0.32 (-0.96; 0.31) | 0.077 |
| HDL-cholesterol (mg/dL) | 56.2 (55.2, 57.2) | 56.2 (52.6, 59.9) | 0.973 | 0.06 (-0.03; 0.14) | 0.04 (-0.28; 0.36) | 0.925 |
| Triglycerides (mg/dL) | 70.4 (66.3, 74.4) | 71.1 (55.1, 87.0) | 0.933 | 0.22 (0.07; 0.37) | 0.24 (-0.34; 0.83) | 0.947 |
| hs-C-reactive protein (mg/L) | 1.75 (1.33, 2.16) | 0.99 (0.20, 2.60) | 0.374 | 0.14 (0.03; 0.25) | -0.05 (-0.46; 0.37) | 0.399 |
| Systolic blood pressure (mmHg) | 104.9 (104.2, 105.6) | 104.4 (102.1, 106.7) | 0.678 | 0.77 (0.70; 0.83) | 0.80 (0.59; 1.00) | 0.770 |
| Diastolic blood pressure (mmHg) | 70.3 (69.7, 70.9) | 67.9 (65.9, 70.0) | 0.030 | 1.14 (1.09; 1.19) | 0.94 (0.76; 1.13) | 0.044 |
| ^a^ Mean values and 95% confidence intervals adjusted for non independence. | | | | | | |
| ^b^ For systolic and diastolic blood pressure - age, sex and height z-score | | |  |  |  |  |
