# Supplementary material for: Different levels of cardiometabolic indicators in multiple vs. singleton children
Source: BMC Pediatr. 2019 Sep 11;19:331. doi: 10.1186/s12887-019-1707-0 (PMC6737661; doi:10.1186/s12887-019-1707-0)
Supplement: Supplementary file 2 — Table S2. Matched pair analysis regarding the comparison between singletons and multiples regarding cardiometabolic indicators at 7 years follow-up evaluation. (DOCX 14 kb) [file 12887_2019_1707_MOESM2_ESM.docx]

| Supplementary table 2. Matched pair analysis regarding the comparison between singletons and multiples regarding cardiometabolic indicators at 7 years follow-up evaluation. | | | | | |
| --- | --- | --- | --- | --- | --- |
|  |  |  |  |  |  |
|  |  |  |  |  |  |
|  | | |  |  |  |
|  | | | **Age and sex z-score^b^** | |  |
|  | | | **Singletons** | **Multiples** | **p** |
| **Cardiometabolic characteristics at age 7^a^** | | |  |  |  |
| Weight (kg) | | | -0.24 (-0.37; -0.12) | -0.42 (-0.54; -0.30) | 0.035 |
| Height (cm) | | | -0.25 (-0.39; -0.12) | -0.45 (-0.57; -0.32) | 0.034 |
| Body mass index (kg/m^2^) | | | 0.46 (0.29; 0.62) | 0.25 (0.09; 0.42) | 0.082 |
| Fat mass index (kg/m^2^) | | | -0.28 (-0.42; -0.15) | -0.41 (-0.53; -0.28) | 0.150 |
| Fat-free mass index (kg/m^2^) | | | 0.07 (-0.08; 0.22) | -0.01 (-0.15; 0.14) | 0.462 |
| Waist circumference (cm) | | | -0.17 (-0.31; -0.04) | -0.41 (-0.53; -0.29) | 0.010 |
| Waist-to-height ratio | | | -0.14 (-0.25; -0.03) | -0.21 (-0.32; -0.09) | 0.417 |
| Glucose (mg/dL) | | | -0.20 (-0.49; 0.08) | 0.13 (-0.15; 0.40) | 0.106 |
| Insulin (μIU/mL) | | | -0.16 (-0.36; 0.05) | -0.12 (-0.31; 0.08) | 0.799 |
| HDL-cholesterol (mg/dL) | | | 0.19 (-0.02; 0.41) | 0.13 (-0.05; 0.31) | 0.635 |
| Triglycerides (mg/dL) | | | -0.17 (-0.36; 0.02) | -0.07 (-0.22; 0.20) | 0.282 |
| hs-C-reactive protein (mg/L) | | | -0.08 (-0.22; 0.05) | -0.10 (-0.18; -0.01) | 0.892 |
| Systolic blood pressure (mmHg) | | | 0.83 (0.70; 0.95) | 0.70 (0.58; 0.83) | 0.148 |
| Diastolic blood pressure (mmHg) | | | 1.12 (1.01; 1.24) | 0.95 (0.87; 1.04) | 0.015 |
| ^a^ Mean values and 95% confidence intervals. | | | | | |
| ^b^ For systolic and diastolic blood pressure - age, sex and height z-score | | | | | |
